# Death of Professor Ed. Jæger

**Published:** 1884-09

**Authors:** 


### Death of Prof. Ed. Jæger.

The city of Vienna, and, indeed, the whole medical world, has to deplore the death of Prof. Ed. Jæger, which occurred July 5th. He was born in Vienna in 1818, and was the son of Frederick Jæger, a famous oculist of that time. Prof. Ed. Jæger was most eminent in ophthalmology, and by one of his pupils was called the master of all the masters in ophthalmoscopy. His reputation was not limited to his own city or pupils, but he was well known abroad for his great scientific attainments and work. His ophthalmoscope, his atlas and his test-types are known to every oculist.

---

We have been favored with a copy of Dr. Charles Parkes' masterly address on the subject of gun-shot wounds of small intestines, read at the meeting of the American Medical Association, last May.

Dr. Parkes' conclusions, based on the results of thirty-seven experiments on the bodies of etherized dogs, the wounds being produced by the ordinary Smith & Wesson revolver, of calibres 22, 32, 38 and 44, and by a 22-calibre rifle, by which one or more perforations of some portion of the intestines were inflicted, are worthy of careful note:

"First. Hemorrhage, following shot wounds of the abdomen and the intestines, is very often so severe that it cannot be safely controlled without abdominal section; it is always sufficient in amount to endanger life by secondary septic decomposition, which cannot be avoided in any other way than by the same treatment.

"Second. Extravasations of the contents of the bowel after shot injuries thereof are as certain as the existence of the wound.

"Third. No reliable inference as to the course of a bullet can be made from the position of the wounds of entrance and exit.

"Fourth. The wounds of entrance and exit of the bullet should not be disturbed in any manner, except to control bleeding or remove foreign bodies when present. They need only to be covered by the general antiseptic dressing applied to the abdomen.

"Fifth. Several perforations of the intestines close together require a single resection, including all the openings. Wounds destroying the mesenteric surface of the bowel always require resection.

"Sixth. The best means of uniting the wounded intestine after resection is by the use of fine silk thread after Lembert's method. It must include at least one-third of an inch of bowel tissue, passing through only the peritoneal and muscular coats, never including the mucous coat. The everted mucous membrane must be carefully inverted, and needs no other treatment.

"Seventh. Wounds of the stomach, small perforations and abrasions of the intestines, can be safely trusted to the continued catgut suture.

"Eighth. Every bleeding point must be ligated or canterized and especial care devoted to securing an absolutely clean cavity.

"Ninth. The best method of treating the stumps of divided mesentery is to save the mesenteric surface of the bowel as above indicated.

"Tenth. *Primary abdominal section* in the mid-line gives the best command over the damage done, and furnishes the most feasible opening through which the proper surgical treatment of such damage can be instituted. Further, its adoption adds but little, if anything, to the peril of the injury.

"Eleventh. Is not the moral effect of the assurance to the patient that he will be placed in a condition most likely to lead to his recovery, a good substitute for the mental depression accompanying the general and popular conviction that these wounds mean certain death?"

Dr. Parkes insists on abdominal section as giving the best chance of controlling hemorrhage, the greatest danger to avoid in injuries of this class. He states that the great tendency to excessive hemorrhage from the severance of even small abdominal vessels is probably due to (*a*) "laxity of tissues through which these vessels course, (*b*) absence of pressure from surrounding soft parts, (*c*) lack of the peculiar influence of the atmosphere, either from its weight or clot-producing power;" and that "these conditions are very quickly altered after air is admitted through the abdominal section. Clots rapidly seal up the smallest vessels; the smaller arteries spurt less forcibly and soon cease beating; the larger ones contract and retract just as occurs in the wounds of soft parts in other regions of the body."

---

THE epitomized article on "Chronic Joint Disease," which will be found among our selections, from a clinical lecture delivered by Dr. Richard Barwell, one of the ablest of English surgeons, emphasizes the great rarity of tubercular diseases of joints in children, and the frequency of occurrence of the strumous or scrofulous habit of body, upon which these joint diseases depend. This is essentially the English view of the question. Tubercle selects rather the internal organs, as, for instance, the lungs, and when found in joints is generally the product of old chronic strumous inflammations, "the terminal and not the initial point of a morbid process." It will be seen from these opinions issued by the highest of English authority, that "the habit of body" is an important factor in the problem. In confirmation of this position the experiments of Schüller in injecting tuberculous matter into lungs of dogs and rabbits, and at the same time injuring their joints, are very important. While as a result of the injections, the internal organs were thickly beset with tubercle, but limited manifestations of tuberculous deposits were found in the injured joints. The author concludes, therefore, that the tuberculous state of body does not produce joint disease. These statements are interesting in view of opinions lately advanced from a local source, that joints were the frequent seat of tuberculous deposits, and that ignipuncture was a successful method of treatment. The English, rather than the more theoretical opinions of the German authorities, coincide with the experience of American surgeons. It would seem that the doctrines of English surgeons should have great weight on this subject, in view of the prevalence of tuberculous disease in that country, and any question that may arise in the profession on the pathology of joint disease would be likely to find in their large and varied experience an authoritative opinion. An eminent writer says that "if we are to restrict the term tubercle to pathological changes in which the tubercle-bacillus is found, then a great number of cases of so-called scrofulous diseases of joints are not tuberculous." We refer to this question at this time in order to correct certain erroneous views which have been advanced on this subject, and to emphasize the conviction that in England, in which tubercle prevails more widely than in any other country, affording the profession a wide and varied experience in its treatment, the sentiments put forth by eminent men should guide American surgeons in their treatment of strumous disease of joints which are more or less prevalent in our large cities.

---

Dr. Cuming, in his address as President of the British Medical Association, at its meeting in Belfast, protests against the tendency to allow the results of anatomical investigation to eclipse the facts gained by clinical observation. The principal interest in regard to the progress of medical science is, as he says, at present concentrated on the study of minute organisms as causes of disease. And while not in the least undervaluing the results obtained by this line of research, he states that their interpretation may be misleading unless viewed in their relation to the clinical phenomena of disease. There are certain racial proclivities and individual tendencies, about which more must be known before we can properly understand the influence of these micro-organisms upon the body. Questions of this kind come within the scope of every practitioner of medicine, while, naturally, the investigation into the part these parasites play in the economy must be reserved for those who have leisure and aptitude for original research. It is in respect to these questions that the best results are looked for from the labors of the Committee for the Collective Investigation of Disease, now actively at work in Great Britain, and Dr. Cuming recommended that an International Committee be formed, to embrace all civilized countries, for the purpose of carrying on this investigation. This is a subject that should interest every physician, and we feel sure that if such a committee were organized, its efforts would be heartily seconded by the profession of this country. The value of a systematic attempt to understand the influence of race and climate, for instance, upon the origin and course of the epidemic diseases cannot be over-estimated. We have particular advantages for this investigation here, on account of the diversity of our people and the variety in our climate, and we trust that an organization for the purpose of carrying out the suggestions of Dr. Cuming will not long be delayed.

---

THE Buffalo Medical College opens its next course Thursday, September 25th. The announcement for the session of 1884-85 furnishes ample information in regard to the facilities offered by this institution. We notice many important changes in its faculty, which we fully commend, noticeable among which is the promotion of its corps of lecturers to the position of professors, a change which it is but justice to these able gentlemen, who have added so much to the reputation of the college, should have been made long ago. The older alumni will miss the honored names of Hadley, Eastman, White and Moore, whose fame has, in the past, given the college much of its reputation. With the stimulus imparted by the events of the past year in the city, we trust we shall witness the inauguration of a graded course of instruction and a higher standard for graduation, innovations in its curriculum, which will place this college *en rapport* with the spirit of the age.

---

THE present number of the JOURNAL receives a fresh impetus from the labors of an able corps of co-laborators, who have undertaken the work of editorial assistance with a view to add new interest to the JOURNAL, and to relieve the editors who are burdened with professional labors which have heretofore interfered with the efficient performance of their duties as journalists.

It is our purpose to make the JOURNAL a faithful exponent of current medical opinions, so that the present volume will prove the most valuable of any in its history. We anticipate ample encouragement from our readers, in frequent contributions and in an enlarged circulation.

---

WE were desirous of making a report of the sanitary condition of this city, but were disappointed on learning, at the office of the Board of Health, that no mortality reports have been made since April 12, 1884. Reports from New York, Albany, Rochester, and even from English cities, could be obtained almost to date; but the Board of Health of Buffalo, from negligence or some other cause, fails to inform the medical profession of the prevailing diseases in the city.

---

WE intended to publish, in this number of the JOURNAL, the proceedings of the meeting of the Erie County Medical Society, held to discuss the cholera question. The absence of the Secretary *pro tem.* from the city delayed the transfer of the minutes to our hands until it was too late for the printer. We hope to furnish a synopsis of the discussion in our next number

